# Validity, reliability, responsiveness and interpretability of the EFAS-DK PROM: an observational cohort study of Danish speaking foot and ankle patients

**DOI:** 10.1186/s41687-025-00897-y

**Published:** 2025-06-13

**Authors:** Mick Nielsen, Jens Kurt Johansen, Anna Kathrine Pramming, Jeannette Østergaard Penny

**Affiliations:** 1 Centre for Evidence-Based Orthopaedics (CEBO), Department of Orthopaedic Surgery, Zealand University Hospital, Lykkebaekvej 1, Koege, 4600 Denmark; 2 Department of Orthopaedic Surgery, Hvidovre University Hospital, Hvidovre, Denmark

**Keywords:** Patient reported outcome measure, PROM, Foot, Ankle, EFAS score, EFAS-DK, Reliability, Validity, Responsiveness, Interpretability

## Abstract

**Background:**

This study is an external evaluation of the Patient Reported Outcome Measure (PROM) EFAS-DK developed by the European Foot and Ankle Society (EFAS). The evaluation included a test of the psychometric properties.

**Methodology:**

From October 2019 to September 2022, 101 patients undergoing elective foot or ankle surgery completed questionnaires (EFAS-DK, SEFAS-DK, EQ-5D-5L) prior to surgery and 6 months post-surgery. A subgroup of patients completed a retest. A foot-healthy group control group was added. Testing covered construct validity with hypothesis testing, floor and ceiling effects, internal consistency (Cronbach’s Alpha), test-retest reliability (ICC 2.1), effect size (ES), Standardized Response Mean (SRM), Smallest Detectable Change (SDC) and Minimal Important Change (MIC).

**Results:**

Moderate construct validity with 59% confirmed hypothesis. High content validity, no floor ceiling effects. Cronbach’s alpha 0.88, ICC 0.93. ES and SRM were both 1.06. SDC 4 and MIC 6. Control group score changes was insignificant.

**Conclusion:**

EFAS-DK is a valid, reliable, and responsive foot and ankle PROM score. EFAS-DK can detect a clinically subjective relevant change score of 6 (25% of the total scale), which makes it useful for implementation in the clinic when evaluating patients undergoing foot and ankle surgery. Comparison with a control group showed results that significantly differ from the patients.

**Level of evidence:**

IIa prospective observational analytic cohort study.

## Abstract (plain language summary)

This study is a validation of a Patient Reported Outcome Measure (PROM) called the EFAS-DK. PROM questionnaires captures a health condition of a patient at a given time without any interpretation of the response by the health professional. PROMs are becoming more widespread in the evaluation of surgical treatments because the treatment effect is best evaluated by the patient themself. EFAS-DK is the Danish version of the EFAS Score, a PROM with the intention to be used globally targeting patients undergoing foot or ankle surgery. The validation process of a PROM intended for clinical use is essential and must be based on the target patient group. A valid PROM must be truthful, reliable and be able to detect relevant score changes. We tested a questionnaire on patients before and after surgery and compared with healthy individuals at similar time intervals. Answers were converted to points and underwent statistical analysis and the questionnaire demonstrated satisfactory results in all aspects of a thorough validation (validity, reliability, responsiveness, and interpretability). With this validated Danish version, we add an acknowledged PROM to clinical daily use for evaluation of foot and ankle patients. Furthermore, it is possible to compare data across borders for scientific purposes.

## Background

According to the Danish national patient register 10,526 adult patients had elective foot or ankle surgeries in 2022.

Patient Reported Outcome Measures (PROM) is important in the evaluation of patients who undergo surgical treatment, as the benefit of a treatment is best evaluated by the patients themselves [1]. A PROM is only an adequate reflection of the treatment effect if it is valid, reliable, and responsive to a change [2].

A previous study found 139 different outcome scales in use in the foot and ankle literature [3]. The AOFAS is the most widely used, but rely on a clinical examination, which excludes the AOFAS from being a PROM [1, 4–6]. The self-reported foot and ankle score (SEFAS) [7, 8] is validated in patients with foot and ankle disorders and translated into different languages [8, 9] including Danish, but not used in many countries. The MOX-FQ is highly suitable for evaluation of foot and ankle surgery [8] but in many cases not free for use. FAOS is validated but only for specific pathologies [1] and contain 42 items which is long in a clinical setting. The FFI has 23 items, but the psychometric properties are not convincing [5].

Aiming for a common score, the European Foot and Ankle Society (EFAS) has developed the EFAS score [10] so far validated in 13 languages. The developmental process was a 3-stage analysis including a principal component analysis (PCA) and an item-response theory (IRT) analysis. These analyses confirmed a single scale 6-item general EFAS score clinical version [11]. Our study began as the Danish validation of the score [12]. The Cosmin guidelines suggests a more thorough validation process [13, 14]. As the EFAS Score protocolled validation system did not report the translation process, include a test-retest procedure or compared to an acknowledged score, we expanded our validation accordingly to conduct a psychometric validation of the score.

We included a control group of foot and ankle healthy individuals to test that the max score indeed was reached in this population as well as testing the score over time and comparing to the results of foot and ankle patients before and after surgery.

## Methods

Adult patients were recruited at two orthopedic foot departments. Patients were excluded if ailments severely affecting walking besides the foot problem or previous surgery in the same foot region were present. They had to read and understand Danish and must be able to complete PROMs without any personal guidance. This Danish research group had the responsibility to include a minimum of 100 patients as the Danish contribution to the EFAS Score validation. Despite researching relevant literature and consulting with the department of biostatistics we could not find a recommended way to estimate sample size. We decided to include 100 patients as required by the EFAS Score committee.

We added a pre-surgical test-retest of the patients enabling for the ideal calculation of test-retest reliability, Smallest Detectable Change, and Minimal Important Change. The test-retest population was a subgroup of the patient group who was continuously selected during initial inclusion of patients. The group was asked to complete the retest 5 days after initial completion.

The healthy control group consisted of hospital staff or familiar persons with no foot or ankle diagnosis record. They were chosen with the intention to be equally distributed based on gender in each decade from twenties to seventies.

### Data generation and handling

Patients completed a pen and paper version of an extended 12 item version of the EFAS-DK questionnaire which was a prerequisite for reporting data to the ongoing EFAS Score validation. This questionnaire also included four separate sports items. This external validation and data analyses process is based on the final 6 item clinical version of the PROM and the anchor question (Section “Minimal important change (MIC)”). EFAS-DK was completed prior to surgery (T0) and six months post-surgery (T2). A subgroup did a retest of the questionnaires after initial completion (T1) to test reliability. The control group completed questionnaires six months apart.

In case of incomplete questionnaires, no guidelines were given by the EFAS committee except if more than one answer were given to an item, the mean was registered. We used the following for all questionnaires; in the case of > 3 unanswered items the questionnaire was disregarded. If the patient had put a mark between to answers, the mean was used. If any discrepancy between the X marked on the EQ-VAS scale and the written box number, the box number was used [15]. For SEFAS-DK in case of one unanswered item the mean of the remaining was registered [7].

Both the EFAS Score and SEFAS were originally validated as a single construct [7, 11]. This validation of the Danish version of EFAS-DK intend to do so as well. However, to maximize the properties of the data (testing for construct validity by hypothesis testing) we made assumptions regarding subscales [8]. That is EFAS-DK item 1,5 and 6 was defined as a pain subscale. Item 2,3 and 4 a physical function subscale. For the 12 item SEFAS-DK item 1, 5, 8, 9, 11, 12 was defined as a pain subscale. Item 2, 3, 4, 6, 7, 10 as a physical function subscale (Table 1).

**Table 1:** Construct validity hypothesis testing

Hypotheses	Correlations	Confirmed
**1.** Correlation between subscales pain (EFAS-DK), pain (SEFAS-DK) and pain/discomfort (EQ-5D-5L) should be ≥ 60% (17)	EFAS-DK vs. SEFAS-DK: 0.66 EFAS-DK vs. EQ5D5L: -0.58	1 of 2
**2**. Correlation between subscales physical function (EFAS-DK), function (SEFAS-DK) and mobility (EQ-5D-5L) should be ≥ 60%	EFAS-DK vs. SEFAS-DK: 0.77 EFAS-DK vs. EQ5D5L: -0.68	2 of 2
**3.** Correlation between total score of the EFAS-DK, SEFAS-DK and EQ-5D-5L ≥ 60%	EFAS-DK vs. SEFAS-DK: 0.74 EFAS-DK vs. EQ5D5L: 0.62	2 of 2
**4.** Stronger correlation between pain (EFAS-DK) and pain (SEFAS-DK) or pain/discomfort (EQ-5D-5L), than pain (EFAS-DK) and physical function (EFAS-DK). The difference should be ≥ 5% (20)	Pain EFAS-DK vs. Pain SEFAS-DK: 0.66 Pain EFAS-DK vs. pain/discomfort EQ-5D-5L: -0.58 Pain EFAS-DK vs. physical function EFAS-DK: 0.75	0 of 2
**5.** Correlation between pain subscales and function/mobility subscales should be ≥ 30% but ≤ 60% (20)(21).	Pain EFAS-DK and physical function EFAS-DK: 0.75 Pain EFAS-DK and physical function SEFAS-DK: 0.57 Pain EFAS-DK and mobility (EQ-5D-5L): -0.54 Physical function EFAS-DK and Pain SEFAS-DK: 0.72 Physical function EFAS-DK and pain/discomfort (EQ-5D-5L): 0.51.	3 of 5
**6.** Correlation between pain EFAS-DK and anxiety/depression (EQ-5D-5L) should be ≥ 30% but ≤ 60%	Pain EFAS-DK and anxiety/depression (EQ-5D-5L): -0.10	0 of 1
**7.** Correlation between EFAS-DK physical function and anxiety/depression (EQ-5D-5L) should be < 45% (22)	Physical function EFAS-DK and anxiety/depression (EQ-5D-5L): -0.15	1 of 1
**8.** Correlation found in (**6**) will be at least ≥ 5% higher than the correlation found in (**7**) (21).		0 of 1
**9.** Effect size (ES) for patients undergoing surgery will be at least 0.80 for the EFAS-DK score indicating large ES.	EFAS-DK ES: 1.06	1 of 1
Summarization of confirmed hypothesis: 10 of 17 hypothesis	59%	

Spearman correlation (baseline data) shown for nine predefined hypotheses across PROMs. EFAS-DK item 1,5 and 6 was defined as a pain subscale. Item 2,3 and 4 a physical function subscale. SEFAS-DK item 1, 5, 8, 9, 11, 12 was defined as a pain subscale. Item 2, 3, 4, 6, 7, 10 as a physical function subscale. Convergent correlation defined as Spearmans ≥ 60%(20)

### Translation and cross-cultural adaption

**Fig. 1:**
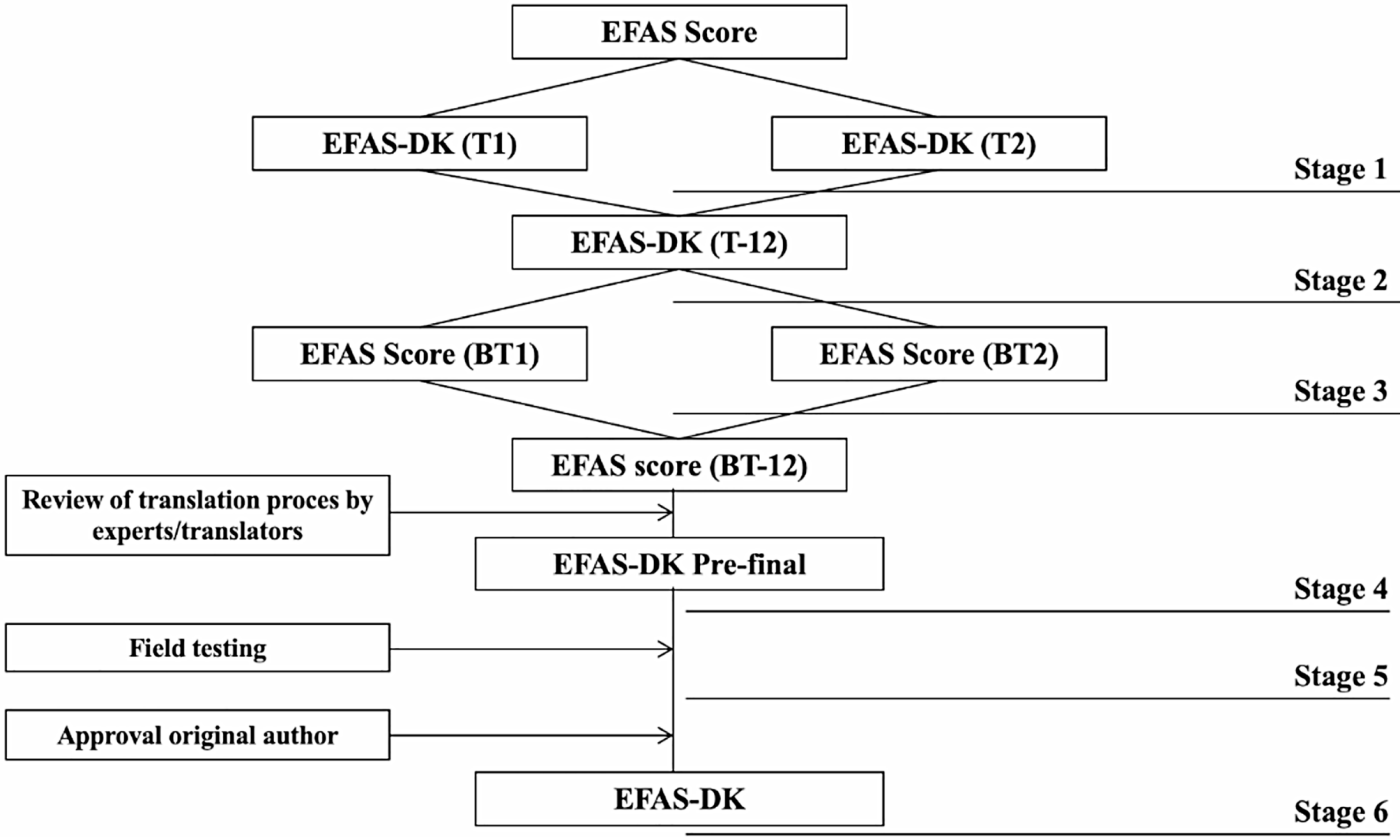
Flow diagram of translation and cross-cultural adaption. Forward translations (T1, T2), synthesis (T-12, BT-12) backward translations (BT1, BT2), Field test of prefinal version (*n* = 16)

Translation and cross-cultural adaptation of the English EFAS questionnaire into Danish (Fig. 1) was based on the guidelines by Beaton et al. 2007 [16]. Two independent forward translations from the original English version into Danish (an orthopedic surgeon and a medical student, both Danish as their mother tongue) (Stage 1), a synthesis version with discussion of discrepancies by the translators (Stage 2). Next was two individual backtranslations. None of the translators had a medical background, both have English as their mother tongue, one is a PhD in English Linguistics, the other a primary school teacher in Danish (Stage 3). Translators were blinded to the original version. The synthesis of the backtranslation was approved by original author. Final review of the process by translators and authors from EFAS-DK and synthesis into Danish prefinal version (Stage 4). The committee, consisting of the research group (medical experts and methodological experts) and translators (language experts) is important in securing cultural equivalence and conceptual equivalence. A test of the prefinal version on 16 hospitalized patients post-surgery from target group (elective foot and/or ankle surgery) noting issues regarding relevance, understanding, formulation or layout of the items or response options (Stage 5). Minor corrections were made by the research group. This contributes to content validity. The revised prefinal version were approved by the original author (Stage 6). By comparing relevant psychometric testing results of the EFAS-DK with the ongoing EFAS Score validation process cross cultural adaption will be evaluated. It follows that cross cultural adaption is secured if the resultant version has sound psychometric properties comparable to the original version.

### Validation process

The study is a prospective observational analytic cohort study [4] with no experimental intervention. The validation is based on patients answering three different PROMs:

**EFAS-DK:** Region specific PROM, validated as a single scale covering pain and physical function. 6 questions, answers based on a likert scale (0–4) with endpoint descriptions. Score range 0–24. A Not Applicable (N/A) box could be chosen for each item. Low scores indicate severe foot disability. The German and Swedish validation found two subscales, pain, and physical function [17].

**SEFAS-DK:** Region specific PROM. 12 multiple choice questions. Answers based on a likert scale 0–4. Score range 0–48. Low scores indicate severe foot disability. The SEFAS is validated as a single scale and covers pain, function, limitation of function [7]. Prior to EFAS-DK, SEFAS-DK was the recommended PROM from the Danish foot and ankle society.

**EQ-5D-5L:** Generic questionnaire EuroQol [15] validated as a measure of health-related quality of life. 5 items comprise five dimensions mobility, self-care, usual activities, pain/discomfort, and anxiety/depression. The score within each dimension ranges from 1 to 5 and creates a 5-digit health state. Bases on a Danish value set the health state is converted into an index value ranging from − 0.757 (worst health state) to 1 [18]. The EQ visual analog scale (EQ VAS) quantifies the patients self-rated health from 0 (worst health) to 100 (best health).

#### Psychometric testing

The psychometric testing is mainly based on the COnsensus-based Standards for the selection of health status Measurement INstruments (COSMIN) guidelines [13, 14]. PROM data was entered fully anonymous in an excel spreadsheet. Statistical analyses conducted by statistician using SAS software.

### Validity

The extent to which a PROM instrument measures the constructs it is intended to measure [13, 19].

#### Construct validity

Construct validity reflects the extent to which a score relates to other scores [17].

##### Hypothesis testing

Hypothesis testing of predefined hypotheses concerning the score of a patient on other instruments or differences between test groups [7, 17]. The a priori hypotheses are devised by the research group based on their expectations of relations between PROM scores and other derived measurements. The definitions of subscales (Table 1) used in the hypothesis testing are based on the research group own assumptions and is intended as a qualified theoretical proposal. ≥75% of the hypotheses should be confirmed if construct validity is present [17, 20], 50% if moderate [17]. Tested by calculating Spearman’s correlation coefficient with correlations ≥ 60 considered strong convergent relations, ≤ 30% as divergent week relation and correlations ≥ 30% but ≤ 60% as mediocre relations [7, 20]. For convergent validity and divergent validity and other known group comparisons, the following relations between PROMs using baseline responses (T0) was made (Table 1). COSMIN consider p-values irrelevant in hypothesis testing [14].

##### Cross-cultural validity

How items of a translated PROM reflect the original items. We compare Effect Size (ES) and Cronbach Alpha (CA)) of EFAS-DK with the original published EFAS Score validations.

##### Structural validity

The degree to which the scores of a PROM are an adequate reflection of the dimensionality of the construct to be measured [13]. The EFAS Score did a developmental three stage process [11] which confirmed the validation of a single scale reflective model. We considered that sufficient.

#### Content validity

The extent to which the content of the items reflects the constructs to be measured [7, 13, 17]. Evaluated by calculating floor/ceiling effects, present if > 15% of the respondents tick of the lowest or highest value of the score-range [4, 7, 17, 23]. Presence indicates a non-comprehensive scale, limiting the reliability and responsiveness because patients cannot be distinguished, and score changes will not be noticed [17].

As mentioned in the translational section “Translation and cross-cultural adaption” content validity is high in the EFAS-DK PROM by following the Beaton et al. guidelines [16] because it ensures consistency and cultural equivalency in the content.

### Reliability

The measurement error [13]. Same as precision/reproducibility [13, 17].

#### Internal consistency

Correlation/interrelatedness between items supposed to measure the same construct [13, 14]. Calculated by Cronbach’s alpha (CA) based on pre surgical EFAS-DK data (T0). 0.70–0.95 is considered good [17]. Alpha > 0.90 might indicate redundancy of items [19].

#### Test-retest reliability

The proportion of the total variance in the measurement, which is because of true differences among patients [13]. The extent to which measurements can be replicated [24]. Calculated as Intraclass Correlation Coefficient (ICC 2.1 (24)), as a 0–1 ratio (19). >0.70 is good [7, 17, 20] > 0.9 is excellent [24]. Visualized by a Bland-Altman plot with 95% Limits Of Agreement (LOA).

#### Smallest detectable change (SDC)

Smallest Detectable Change (SDC) is the minimum intraindividual change above the threshold of the standard error of the measurement (SEM) of the ICC [8]. Calculated using a distribution-based approach as the half width of the Limit Of Agreement (LOA) interval based on test-retest data.

$$\:LOA={Mean}_{T1-T0}\pm\:1.96*{SD}_{change}$$

$$\:SEM=\frac{{SD}_{change}}{\sqrt{2}}$$

$$\begin{aligned}\:SDC &= 1.96*\sqrt 2 *SEM \hfill \\ &= 1.96*\sqrt 2 *\frac{{S{D_{change}}}}{{\sqrt 2 }} \hfill\\&= 1.96*S{D_{change}} \hfill \\ \end{aligned} $$

### Responsiveness

The ability of a PROM to detect change in score due to an intervention [7, 14, 17].

#### Effect size (ES)

Effect size (ES) is the score difference T2-T1, divided by the standard deviation (SD) at baseline [11, 23]. ES > 0.8 considered large [7, 23], ES = 0.5–0.8 moderate [25]. COSMIN checklist states ES as inappropriate [14]. ES will be calculated for comparison with ES of the ongoing multilingual EFAS Score validations. This contributes to the cross cultural- and Criterion validity.

#### Standardized response mean (SRM)

Standardized response mean (SRM) is the score difference T2-T1 divided by the standard deviation of the change [7, 8, 23]. We used the same reference values as for ES.

### Interpretability

The degree to which one can assign qualitative meaning to quantitative scores. Interpretability is not considered a measurement property according to COSMIN guidelines [13]. The research group choose to include this key concept, nevertheless.

#### Minimal important change (MIC)

Minimal Important Change (MIC) is estimated using an anchor-based approach which is generally preferred [2]. MIC is the smallest change in score, perceived as clinically relevant by the patients [25]. As an anchor we added an extra item EFAS-DK Q7 “Have you improved after surgery” which is a supplemental question where patients at T2 is questioned how they rate themselves after surgery relative to T0 by answering on the 0–4 Likert scale. We further compare the MIC to SDC, to confirm that we can detect the MIC within a patient, comparing pre- and post-surgery data in the EFAS-DK. MIC is only detectable and relevant if MIC > SDC. MIC was defined as mean change (median) score in patients who answered 3 on the 0–4 Likert scale.

## Results

**Fig. 2:**
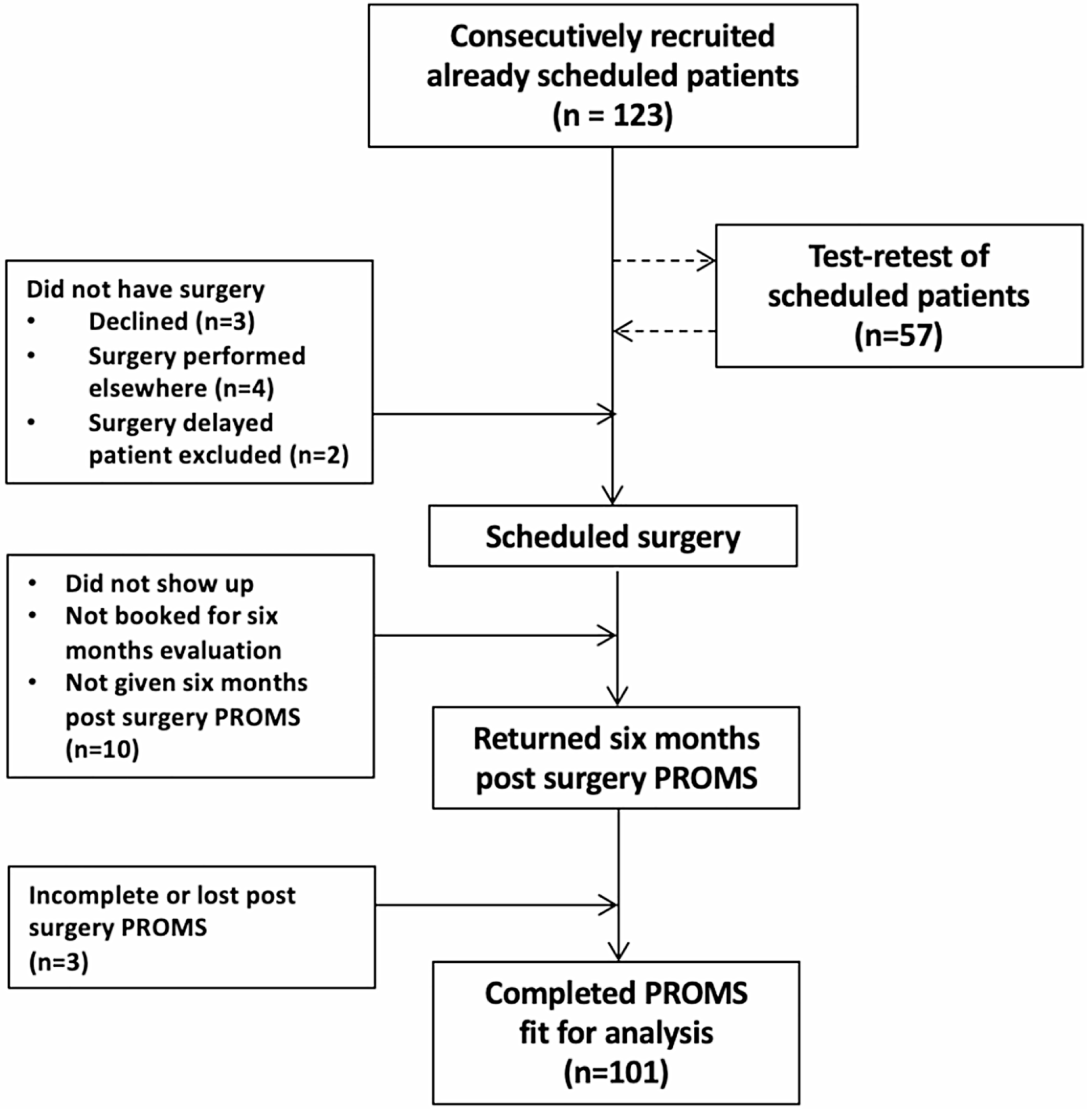
Flow diagram of recruitment and exclusion of patients. 101 patients collectively contributed to the data pool. Stratified on PROMs 100 patients completed EFAS-DK (81,3%) and 95 patients completed SEFAS-DK and/or EQ5D5L (77,2%). A subgroup of 57 patient did a test retest of the PROMs

From October 2019, we consecutively recruited 123 patients. 101 patients had contributed to the data pool (Fig. 2). 100 of the patients (81.3%) completed EFAS-DK at T0 and T2, 95 of the patients (77.2%) completed SEFAS-DK and/or EQ5D5L at T0 and T2 (for explanation that leaves one patient who completed SEFAS-DK and EQ5D5L but not EFAS-DK). To secure data all patients was scheduled to a follow up consultation six months post-surgery regardless the need. 57 patients did a retest 5.5 days after initial completion (T1). A total of 66 individuals were recruited for the control group, of whom 48 completed the questionnaires sufficiently (Table 2). 45 persons completed the EFAS-DK and SEFAS-DK whereas 47 completed the EQ5D5L.

**Table 2:** Demographic data of patients, test-retest (subgroup of patient group), and controls

Demographics	Patients	Test-retest	Controls
Cases	101	57	48
Age mean years (median)	56.4 (59)	55.1 (56)	50.5 (52)
Age min/max years	18–84	20–83	21–77
% women	66%	71%	51%
Mean T1-T0 (days)	–	5.5	–
Mean T2-T0 (days)	260	–	197
Mean T2- day of surgery	185	–	–
Forefoot, midfoot, hindfoot %	26.7/7.9/17.8	26.3/5.3/14.0	–
Ankle, flat or cavus foot %	25.7/21.7	28.1/7.0	–
Other %	15.8	16.3	–
Osteoarthritis (M19) %	33	28	–
Deformities (M20-21, Q66) %	41	40	–
Soft-Tissue (M60-79) %	5	5	–
Other musculoskeletal (M) %	4	4	–
Other Diagnosis %	18	23	–

T0: completion of questionnaires at consultation before surgical treatment, T1: retest of the questionnaires after initial completion but before surgery, T2: completion of questionnaires post-surgery consultation

### Validity

#### Construct validity

Nine predefined hypothesis (Table 1) generated 17 hypotheses relevant for EFAS-DK. As mentioned in the method section “Hypothesis testing” the definitions of subscales are based on our own assumptions. EFAS-DK and SEFAS-DK is validated as a single scale. 59% of the hypothesis was confirmed indicating moderate construct validity. There was a strong correlation (≥ 0.60) i.e. convergent validity between EFAS-DK and SEFAS-DK total score, physical function, and pain subscales. Solely looking at hypothesis 3 and 9 regarding total scores (i.e. single scale) 3 of 3 generated relevant hypotheses for EFAS-DK was confirmed (Table 1). Divergent correlation of -0.10 was showed between Pain EFAS-DK and anxiety/depression (EQ-5D-5L) (hypothesis 6) and − 0,15 between physical function EFAS-DK and anxiety/depression (EQ-5D-5L) (hypothesis 7).

#### Content validity

Floor ceiling effects was evaluated in 95–100 patients (numbers varies amongst PROMs) at baseline. We found no floor or ceiling effect in any PROM (Table 3) even after stratifying for hypothetical subscales.

**Table 3:** Responsiveness, interpretability, and floor ceiling effect for patients and control group

	T_0_ mean (SD)	T_2_ mean (SD)	ES (95%CI)	SRM (95%CI)	Floor/ceiling (%)	SDC	MIC (median)
EFAS-DK (*n* = 100)	8.5 (5.3)	14.1 (5.5)	1.06 (0.74;1.38)	1.06 (0.81;1.31)	2/1	4.0	6.5 (6)
SEFAS-DK (*n* = 95)	22.4 (9.1)	32.8 (9.6)	1.15 (0.82;1.49)	1.1 (0.84;1.35)	0/0	4.9	10.6 (11)
EQ5D5L index (*n* = 95)	0.6 (0.7)	0.8 (0.2)	-0.82 (-1.1;-0.54)	0.79 (0.55;1.02)	1/1	0.28	–
EQ-VAS (*n* = 95)	68.6 (20.9)	73.1 (17.6)	0.21 (-0.05;0.48)	0.21 (0.0072;0.42)	1/3	6.3	–
EFAS-DK control (*n* = 45)	22.84 (1.81)	22.38 (2.48)					
SEFAS-DK control (*n* = 45)	46.53 (2.11)	46.12 (3.41)					
EQ5D5L index control (*n* = 47)	0.98 (0.051)	0.94 (0.19)					
EQ-VAS (*n* = 47)	89.13 (12.37)	47.57 (12.09)					

Numbers of participants (n) shown in parentheses in left column

### Reliability

#### Internal consistency

Cronbach’s Alpha was 0.88 for EFAS-DK (Table 4). No item is redundant. For SEFAS-DK it was 0.87. CA for the EFAS-DK control group was 0.70 which is significantly lower than for the study population, indicating that the PROM is designed for the target population. Mean European CA is 0.81 (13 languages, range 0.86–0.92, median 0.86).

**Table 4:** Reliability

PROM	T_0_ mean (SD)	T_1_ mean (SD)	α	ICC
**EFAS-DK** (*n* = 57)	8.89 (5.39)	8.97 (5.66)	0.88 (*n* = 100)	0.93
Pain	4.39 (2.70)	4.48 (2.96)	0.81	–
Physical function	4.50 (3.00)	4.49 (3.02)	0.78	–
**SEFAS-DK** (*n* = 52)	24.27 (9.32)	24.62 (9.46)	0.87 (*n* = 95)	0.97
Pain	9.42 (4.14)	9.49 (4.10)	0.80	–
Physical function	14.85 (5.74)	15.13 (5.96)	0.76	–
**EQ5D5L index** (*n* = 54)	0.65 (0.26)	0.64 (0.27)	–	0.85
**EQ-VAS** (*n* = 54)	72.33 (18.72)	71.19 (18.84)	–	0.98

Reliability test for all PROMs. For EFAS-DK and SEFAS-DK hypothetical subscales pain and physical function is also shown. Test-retest reliability (ICC) and internal consistency (Cronbach’s alpha α). Numbers of participants shown in parentheses (n)

#### Test-retest reliability

ICC was 0.93 for EFAS-DK (Table 4). It means that 93% of the variation across the test-retest is because of difference between patients. 7% of the variation is difference within answers of the individual patient. ICC 0.97 for SEFAS-DK, 0.85 for EQ5D5L and 0.98 for the EQ-VAS scale. All indicating excellent test-retest reliability.

#### Smallest Detectable Change (SDC)

SDC was 4 (Table 3). A change in EFAS-DK above 4 is a real change with 95% confidence level for the individual patient given that the differences follow a normal distribution. For SEFAS-DK SDC was 4.9 (10.2%).

**Fig. 3:**
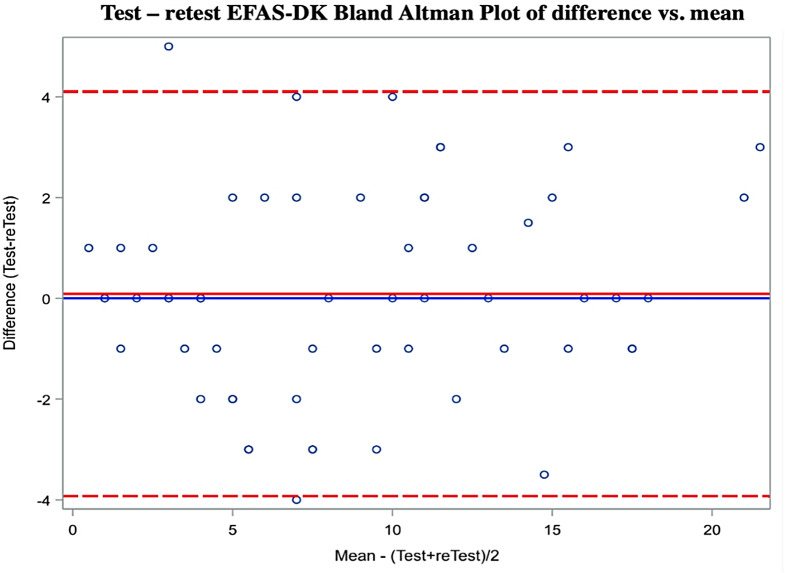
Bland Altman plot for EFAS-DK test-retest total score. LOA is a projection of SDC. Red dotted lines showing 95% LOA interval. Black line is mean difference (T1-T0). Each dot represents a single patient (*n* = 100)

Bland Altman plot for EFAS-DK test-retest shows no systematic error with a mean difference close to zero (0.08) and values within LOA (Fig. 3).

### Responsiveness

Mean change in EFAS-DK scores from T0 to T2 for patients was 5.6 (*p* < 0.0001 paired T test). Change score in all PROMs was statistically significant (*p* < 0.05).

**Fig. 4:**
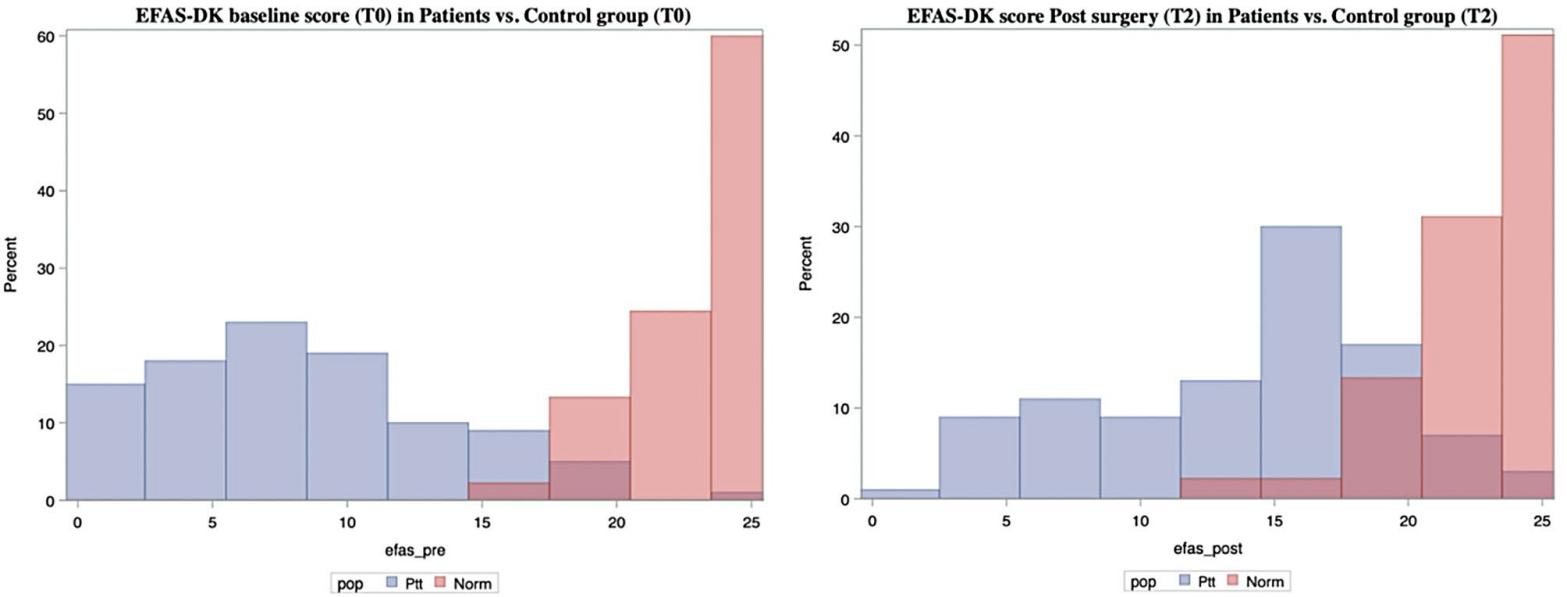
Graphic presentation of T0 and T2 (post-surgery) score for patients (Ptt shown in blue) vs. T0 and T2 score for controls (Norm shown in red)

**Fig. 5:**
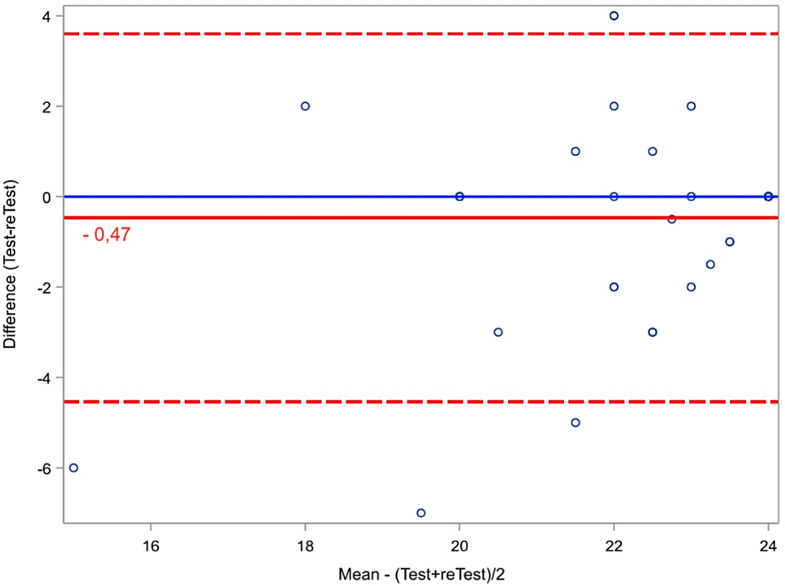
Bland Altman plot with LOA for control group (*n* = 48) T2 vs. T0. Red line is mean change − 0,47. Dotted red lines shows LOA

#### Effect size

EFAS-DK ES was 1.06 (0.74–1.38) (Table 3). Mean change in EFAS-DK score from T0 to T2 for all patients increased with 1.06*SD_Baseline_ i.e. 5.6. This should be seen in the perspective that 2/3 of all patients were within 8.5 ± 5.3 at baseline. SEFAS-DK ES was 1.15 (0.82–1.49). Mean European ES was 1.09.

#### EFAS-DK SRM

SRM was 1.06 (0.81–1.31). Mean change from T0 to T2 was 5.6 for the EFAS total score. This should be seen in the perspective that 2/3 of the patients had a change with this mean ± SDC_Change_ i.e. 8.5 ± 5.3. For SEFAS-DK SRM was 1.10 (0.84–1.35). By chance ES and SRM are identical is this case.

### Interpretability

#### EFAS-DK MIC

EFAS-DK MIC was 6.45 (median 6). This result means that we conclude that the true subjectively relevant score change is 6 (25% of the total scale) (Table 3). Data is from the 23 patients answering 3 in Q7 (anchor question). Every increase of one on the 0–4 likert scale correspond to a change in EFAS-DK score of 2.19. These results are further supported by the lack of floor/ceiling effects. SEFAS-DK MIC was 10.6 (median 11) (23% change of the total scale). Number of patients with change scores exceeding MIC i.e. responders was 42 for both PROMs.

## Discussion

EFAS-DK results showed strong associations with SEFAS-DK and EQ5D5L which confirms a strong developmental foundation of the 6-item single scale EFAS Score by the original authors [11]. The moderate construct validity most likely reflects that the test of predefined hypothesis was hypothetical and based on our own assumptions. Definitions of predefined hypotheses was difficult because all three PROMs are validated as a single scale. The results concerning subscales should be interpreted with cautiousness. Despite a comprehensive psychometric testing with satisfying results a one scale PROM limits the use in the clinic because it is unable to point out in which domain (e.g. pain, function, limitation) the patient experiences issues. It is a drawback but the price for a quick and simple score. As stated in the German external evaluation [6], and we agree that the EFAS Score is useful in evaluating the effect of surgical intervention. Similar ES and CA was found in European EFAS validations (mean values) and EFAS-DK. This confirms a successfully translated and culturally adapted PROM [16] and suggest high criterion validity and content validity. The cultural differences between Denmark and England [26] as well as other northern European countries [27] is considered to be relatively small. The items translated did not contain national tradition issues or characteristics which could be concerning talking about cross cultural adaption [28]. The EFAS Score committee who originally developed and validated the EFAS Score PROM [11] originally came from different countries of Europe. 48% of the authors came from northern Europe.

This validation process did not include a new evaluation of structural validity but rely on the strict cross cultural translational process and calculation of Cronbach’s alpha as an indirect indicator of structural validity.

The strength of this study is the test-retest and addition of a control group, which highlights differences in scores. It gives an indication of differences between the scores of patients with foot and ankle disease and a healthy control group which to our concern has not been done before. The control group matched the patient group on age (unpaired two sample t-test, *p* = 0,07) and gender (Chi Square test, *p* = 0,60). Difference in follow up was about two months. Based on author recruitment one could argue that controls might belong to a higher social class than patients which carries a risk of bias in baseline values for the controls, but it should not affect the conclusions.

Mean score change in EFAS-DK for control group was insignificant. Both at baseline and post-surgery, score data from patients are quite different from controls (Fig. 4) which shows that the PROM is sensitive to change after intervention. Whether the change in scores is driven by the surgical intervention cannot be unambiguously concluded because a randomized control trial (RCT) cannot be performed. Comparing Bland Altman plots for test-retest for patients (T0 and T1) and controls (T0 and T2) (Fig. 3 vs. Figure 5) shows that the scores of the healthy control group lies as stable as the patients despite the big time span in test.

MIC > SDC for EFAS-DK and SEFAS-DK confirms the usefulness of the PROM in this population. SEFAS-DK is more sensitive to detect a statistical change (SDC) in scores than EFAS-DK (16.7% vs. 10.2%). No difference between a clinical change (MIC) could be found. A 25% EFAS-DK score change was significant. EFAS-DK is shorter and assumingly faster to complete. A MIC value of 6 for EFAS-DK correspond to findings in an external German EFAS validation [6] which is also the first external evaluation of the EFAS Score. The use of an anchor-based method for MIC calculation is a further strength of our study. The EFAS-DK MIC data is based only on answers from the 23 patients answering 3 on the PGA scale. The amount of data might seem low but including data from patients answering 4 on the PGA scale would cause selection bias because these patients is expected to have an improvement in foot/ankle health. The MIC value is not immutable. It may or may not vary across (sub)populations, different diagnosis and/or treatments [29, 30]. An analysis on MIC across subgroups could not be performed because subgroups would be too small. With the large data amount continuously gained by the EFAS Score validation it might be possible to investigate if and how the MIC varies among subgroups.

Our cohort is heterogeneous. It could be a strength but also a limitation. Subgroups of patients based on ICD-10 diagnosis is relatively small on numbers mainly consisting of patients with osteoarthritis and deformities but relatively spread out on foot region (Table 2). A way to increase the statistical foundation aiming to make assumptions about treatment effects within a large population with several different diagnosis is to increase sample size.

Based on our good floor-ceiling results, the face value of content validity is high. A draw back of the underlying original EFAS score is that it is investigator chosen rather than based on focus group interviews [17]. The process of reducing 169 items from existing questionnaires to 38 items in stage two is not transparent. Nor is it clear how the rating of relevance of the item by patients [11] has on the final choice of items.

When using the EFAS Score in clinical setting a guideline on how to handle N/A answers was not given by the EFAS. This must be addressed from the EFAS Score committee to ensure identical completion of the PROM nation- and worldwide. There is a risk of bias from exclusion or loss to follow up. We had excellent test-retest results but 5.5 days between tests carries a risk of recall bias [17].

## Conclusion

Satisfactory psychometric testing results was reached. This validated EFAS-DK PROM will form the basis of the implementation of a reliable PROM suitable for the evaluation of elective surgery on danish foot and ankle patients. It is short, easy to use and faster to complete than most other relevant PROMs. This is essential for proper implementation in the daily clinical setting for both doctor and patient. The implementation of the EFAS Score in Denmark and other countries is supported by the relevant foot and ankle society in the given country. The implementation of EFAS-DK is supported by the Danish Foot and Ankle Society (DFAS) forming the foundation of its usage on a national scale. The multilingual validations of the EFAS Score makes it easier to compare or gather research results across countries.

## Data Availability

The datasets generated and/or analyzed during the current study are not publicly available due to local data protection regulation but are available from the corresponding author on reasonable request.

## Abbreviations

PROM: Patient Reported Outcome Measure
EFAS: European Foot and Ankle Society
EFAS-DK: Danish version of EFAS Score
SEFAS: Self-reported foot and ankle score
EQ5D5L: EuroQol Group instrument containing 5 dimensions and 5 levels
EQ VAS: EuroQol Visual Analogue Scale
